# Dual Career Development Perspective: Factors Affecting Quality of Post-sport Career Transition of Employed Olympic Athletes

**DOI:** 10.3389/fpsyg.2021.800031

**Published:** 2022-01-04

**Authors:** Petra Robnik, Edvard Kolar, Boro Štrumbelj, Marko Ferjan

**Affiliations:** ^1^Organization and Management of Human Resources and Educational Systems, Faculty of Organizational Sciences, University of Maribor, Kranj, Slovenia; ^2^Athletes Career Centre, Olympic Committee of Slovenia, Ljubljana, Slovenia; ^3^Laboratory of Biodynamics, Faculty of Sport, University of Ljubljana, Ljubljana, Slovenia

**Keywords:** dual career, Olympic athletes, post-sport career transition, factors of development, employment, quality

## Abstract

Although Olympic athletes are celebrated for their sports achievements, they often face serious difficulties in their post-sport career employment. Factors of development that are affecting the quality of post-sport career transition of Olympic athletes are important to acknowledge in the dual career (DC) development perspective. Due to the side lining of academic activities, athletes are often not well prepared for the labor market. If they do not gain sufficient financial background in their careers, it can lead to a lack of proper economic inclusion of athletes in their post-sport career employment and further impact their lives. Career transitions of athletes have been the subject of research in different aspects of DC support (e.g., athletic, psychological, psychosocial, academic/vocational, financial), but most research is linked to the student-athlete DC perspective. Therefore, the aim of our research was to examine the impact of factors directly contributing to the quality of the post-sport career transition in Slovenian elite and Olympic athletes and the social class position and employment of these athletes after the termination of their sports career. From DC support practice, we learned that although athletes often have a proper level of education, their post-sport career transitions were not successful. To fill this gap, 168 elite athletes (M_age_ = 33.34, SD = 13.1) from Slovenia were asked to complete online questionnaires. The results showed a significant contribution of education and DC support-related finances (e.g., employment of athletes in public administration) to the quality of post-sport career transition. Regarding developing a national DC model and based on empirical research, this study identifies the social class position and employment status of former elite athletes from Slovenia. It also identifies opportunities for further research on the quality of the post-sport career transitions and perspectives on DC support. Understanding how different factors contribute to the integrated development of individual athletes to reach their potential in sports, education, and their post-sport career employment is important for theorists, DC practitioners, and stakeholders working with DC athletes. To develop a sufficient mechanism, DC support providers should consider supporting education along with the financial support of athletes during their sports careers and recognizing study-training ecosystems, based on good practices to successfully transition to their post-sport careers. These findings can also be useful for athletes and their athletic triangle support network (e.g., coaches and parents) as a support in the decision-making.

## Introduction

The dual career (DC) of athletes is crucial for the integrated development of individual athletes in sports, education, and post-sport career employment. According to the European Commission orientations (European Commission and Sport, 2016) regarding the minimum quality requirements, the DC of athletes is a complicated political sphere that connects several interesting aspects (i.e., education, young people, health, and labor market) with the joint effort to direct athletes to appropriate career development. A successful DC can result in the lifelong excellence of athletes. The DC framework of athletes includes professional planning and organization of optimal conditions for their integrated development. A growing trend in recent research on athletes’ DC also shows interest in studying their support system (e.g., coaches, parents, peers, educators, support staff members) and their perspectives on DC regarding their support for education and work (Stambulova and Wylleman, 2019). The importance of parents and coaches’ support for athletes’ DC is illustrated in the athletic triangle, with athlete-parents, athlete-coaches, and coach-parents relationships (Wylleman and Lavallee, 2004; Hakkers, 2019). Most recent research (Geary et al., 2021) outlined a case for change in how we refer to DC athletes as a person first approach. DC athletes combine education, training, or work with a sporting career within broader sports management and educational environments. This study examines the literature regarding DC athletes’ stereotypes, labeling, identity, and wellbeing and proposes a change in how we refer to people engaging in DC.

Sport achievement is the basis for DC support and athletes’ status rights. According to the European Guidelines on Dual Careers of Athletes (European Commission [EC], 2012), a talented athlete is recognized by a sports organization as having the potential to develop an elite sporting career. Elite athletes are those who have a professional contract with a sports employer or organization and/or have a recognized status as an elite athlete such as Olympic athletes. When providing DC support, more attention is given to special target groups (i.e., winners of Olympics, world, and/or European championships), but it is important to establish equal possibilities for elite athletes. Many elite athletes have difficulties in the transition to post-sport career employment and proper inclusion in the normal life environment (e.g., regular job, family, etc.) (Stambulova, 2003). Previous studies suggested that demographic aspects such as age, sex, and type of sport have not shown clear associations with the quality of adjustment to post-sport life (Cecić Erpič et al., 2004). However, research (Park et al., 2013; Ryba et al., 2021) has shown sex, educational status, marital status, and competitive levels have an influence. The reasons for sports career termination (Küttel, 2017) can be related to athletes’ jobs, education, performance, sport environment, health, family, finance, personal reasons, and motivation. Concerning the educational status, previous studies underlined that the educational achievement of athletes positively affects the transition quality of elite athletes (Cecić Erpič et al., 2004; Guidotti et al., 2015) and influences the retirement processes (Tshube and Feltz, 2015).

Career transition out of elite sports is a dynamic, multidimensional, multilevel, and multifactor process in which nationality and culture play a crucial role in the post-sport career (Alfermann et al., 2004). Various approaches have been used to study post-sport career transitions. For instance, one study (Martin et al., 2014) tracked changes in the athletic identity and life satisfaction of elite athletes over time as a function of retirement status and voluntariness of retirement decisions. Another study (Knights et al., 2016) presented a systematic review of the literature investigating factors affecting the successful end-of-career transition among elite athletes. Küttel et al. (2017) compared the athletic retirement of former Swiss, Danish, and Polish athletes and explored the influence of factors on the quality of the post-sport career transition. Based on existing transitional models (Park et al., 2013), they developed a model to investigate the predictive power of commonly assumed resources and barriers related to the post-sport career transition. The study criteria were participation in competitions at the international level, carded by a national elite sport governing body, and retired 1–5 years before data collection. Following the previously established models (Stambulova, 2003; Wylleman and Lavallee, 2004; Wylleman et al., 2013), athletes’ career transition can be understood as critical periods between different phases of the athletic, psychological, psychosocial, academic/vocational, and financial level of their career. Based on this understanding, we assumed that 5 years after the sports career termination is a sufficient period for athletes to observe progress in their post-sport careers and to (a) reassess values, (b) choose a new area of operations, and (c) show positive results in their post-sport career employment. To measure the socioeconomic status of retired elite athletes in their post-sport careers, we followed the social-class concept (Erikson and Goldthorpe, 1992; Smith et al., 2013). This concept is complex enough to present a comprehensive picture of an individual on a hierarchical level within society, identify individuals with employment status, answer questions about equality, and indirectly reveal the financial-material situation, and it is largely associated with acquired qualifications. The social-class concept refers to education.

To summarize, further examination of factors that enable a successful DC (i.e., sport and education) and directly contribute to the quality of post-sport career transitions of elite athletes is relevant and important. Understanding how different factors help individual athletes reach their potential in sports, education, and their post-sport career employment is a critical issue for theorists and DC practitioners and stakeholders working with the complexities of this area. Specifically, this information is important during athletes’ decision-making process. Thus, the research aimed to examine (a) the impact of factors contributing to the quality of the post-sport career transition in Slovenian elite and Olympic athletes and (b) the social-class position and employment status of these athletes after their sports career termination. Most previous DC research addressed the study-sport combination, but there is a lack of studies on the work-sport combination (Stambulova and Wylleman, 2019; Fuchs et al., 2021; Moreno et al., 2021). Some studies (Vickers and Morris, 2021) suggest there is no research focusing on athletes’ pathways after graduating from a university and the factors underpinning their decisions. Therefore, studies focus on athletes’ DC and factors in this context but do not directly investigate them regarding the performance of athletes in post-sport career employment. As authors (Zafeiroudi et al., 2020) suggested, based on their understanding of career development learning, which relates to learning about the content and the process of career development of life (McMahon et al., 2003), an athletic career appears to be just one part of the life career, which concerns athlete’s future when retiring from the sport to be well prepared for development in other spheres. DC support practice in Slovenia shows that although athletes often have the appropriate education level, which directly shows successful DC mechanisms in education, it does not guarantee successful employment. Therefore, we focused our study on employed elite and Olympic athletes to be able to research the post-sport career transition quality and the employment status of these DC athletes. Concerning the relationships between factors contributing to the quality of post-sport career transition consistent with empirical research (Küttel, 2017; Küttel et al., 2017), we hypothesized that (a) levels of education, (b) sports achievements, (c) DC support, and (d) reasons for sports career termination have a significant influence on the social-class position of athletes and their post-sport career employment.

## Materials and Methods

### Participants

A sample of 168 athletes (M_age_ = 33.34; 102 men and 66 women) voluntarily participated in the study. Most were competitive elite and Olympic athletes (*n* = 96). Competitive elite athletes are athletes who are competing at the international level, carded by a national elite sport governing body, and are active in their sport careers (Wylleman et al., 2013). The sample included 72 retired elite and Olympic athletes. Since we assumed that 5 years after the sports career termination is a sufficient time for athletes to observe progress in their post-sport career employment, we considered the limitations that athletes (a) have retired more than 5 years before data collection (*n* = 72) and (b) were employed to answer the questionnaire regarding their social-class position. From the sample of 72 retired elite and Olympic athletes, we searched for employed athletes. This resulted in a breakdown of our sample to 49 (*n* = 49) retired elite and Olympic athletes from Slovenia (M_age_ = 44.27, SD = 11.36; 33 men, 16 women) who were employed in their post-sport career and further investigated in this study. The sample was selected through a non-random sampling including all areas of Slovenia (Daniel, 2011). A summary of participant characteristics is presented in Table 1. In the overall sample (*n* = 168), 11 athletes were in winter sports disciplines, of which 10 were part of the Olympics (e.g., winter skiing disciplines; *n* = 57; 34%). Moreover, 23 were in summer sports disciplines, and of them, 20 were in the Olympics (e.g., handball; *n* = 29; 17% and basketball; *n* = 17; 10%). Other sports disciplines were represented by fewer than 10 responses (*n* = 65; 39%). During their athletic career, former elite athletes (*n* = 49) were included in different sports disciplines, from the individual sports (e.g., alpine skiing, athletics, biathlon, snowboarding, gymnastics, judo, kayak-canoeing, cycling, Nordic combined, ski jumping, cross-country skiing, sport climbing, taekwondo, and rowing) to the team sports (e.g., ice hockey, volleyball, and handball). A heterogeneous sample was selected from various individual and team sports, male and female athletes, to maximize the external validity and generalizability. We are aware that the sample size is a limitation of our study. However, participants are elite and Olympic athletes, and the sample is comparable to other similar studies on elite athletes’ DC (Tshube and Feltz, 2015; Defruyt et al., 2020; Moreno et al., 2021; Vickers and Morris, 2021).

**TABLE 1:** Summary of participant characteristics.

Participants characteristics	Classification	Frequency (*n*)	Mean (SD)
Sample characteristics	Elite and Olympic athletes in SI (population size) Sample of elite and Olympic athletes (participants) Competitive elite and Olympic athletes Retired elite and Olympic athletes Retired elite and Olympic athletes–employed* elite and Olympic athletes	533 168 96 72 49	
**Elite and Olympic athletes (*n* = 168)**			
Sex	Female athletes Male athletes	66 (39%) 102 (61%)	
Age			33.34 (13.09)
Athletes career length			11.54 (6.49)
Highest education level			4.96 (1.72)
	Less than primary school	0 (0%)	
	Primary school	13 (8%)	
	Vocational school (2/3 years)	8 (5%)	
	Secondary school	76 (45%)	
	College (university)	5 (3%)	
	Technical college, the first Bologna cycle	18 (11%)	
	University degree or second degree Bologna (Bologna master’s degree)	40 (24%)	
	Master’s degree	5 (3%)	
	Doctorate of science	3 (2%)	
Sport disciplines	Number of sport disciplines athletes included	34	
Elite and Olympic Athletes Status (SPO)	Olympic-class athlete, world-class athlete, international-class athlete, perspective-class athlete, and national-class athlete	28 (18%) 51 (33%) 57 (37%) 11 (7%) 21 (5%)	2.5 (1.1)
**Retired elite and Olympic athletes—employed (*n* = 49)**			
Sex	Female athletes Male athletes	16 (31%) 33 (69%)	
Age			44.27 (11.36)
Athletes career length			14.45 (4.63)
Highest education level			6.14 (1.47)
	Less than primary school	0 (0%)	
	Primary school	0 (0%)	
	Vocational school (2/3 years)	1 (2%)	
	Secondary school	11 (22%)	
	College (university)	2 (4%)	
	Technical college, the first Bologna cycle	7 (14%)	
	University degree or second degree Bologna (Bologna master’s degree)	24 (49%)	
	Master’s degree	2 (4%)	
	Doctorate of science	2 (4%)	
Sport disciplines	Number of sport disciplines athletes included	17	
Elite and Olympic athletes status (SPO)	Olympic-class athlete World-class athlete International-class athlete Perspective-class athlete National-class athlete	10 (20%) 16 (33%) 19 (39%) 3 (6%) 1 (2%)	2.37 (0.95)

*Required sample size: n = 47 (margin of error 5%; confidence level 95%).

### Measures

A previously validated social class scheme (CASMIN version) (Erikson and Goldthorpe, 1992; Evans and Mills, 2000; Smith et al., 2013) adapted from Goldthorpe was used to measure the social-class position of former elite athletes 5 years after their sports career termination (CLASS). The CLASS was used for empirical research to determine the dependent variable’s value. It consists of nine levels of measuring the social-class position of individuals in society, namely, 1. (I) higher-grade professionals, administrators, and officials; managers in large industrial establishments; and large proprietors; 2. (II) lower-grade professionals, administrators, officials, and higher-grade technicians; managers in small industrial establishments; and supervisors of non-manual employees; 3. (IIIa) routine non-manual employees and higher-grade employees (administration and commerce); 4. (IIIb) routine non-manual employees and lower-grade employees (sales and services); 5. (IVa) small proprietors, artisans, etc., with employees; 6. (IVb) small proprietors, artisans, etc., without employees; 7. (IVc + V + VI) skilled workers and small-time owners; 8. (VIIa) semi-skilled and unskilled workers; and 9. unemployed. Adjustments were made as there was no category for the unemployed. However, semi-skilled and unskilled workers were specifically categorized according to economic activities (such as agriculture), whereas the number of persons employed in agriculture in Europe is reducing (Smit et al., 2020). Participants self-evaluated their CLASS regarding the work category in their post-sport career employment, where (1) represents the highest social class (i.e., officials and managers) and (9), the lowest social class (i.e., unemployed).

The definition of social classes also refers to education (EDU). Therefore, according to previous studies (Cecić Erpič et al., 2004; Guidotti et al., 2015; Tshube and Feltz, 2015), athletes’ education levels were measured. For this, the standard classifications of education in Europe were used (Küttel et al., 2017; European Commission [EC], 2018). Participants self-evaluated their educational levels achieved on a nine-level scale, namely, (1) less than a primary school, (2) primary school, (3) vocational/technical school (2/3 years), (4) secondary school, (5) college (university), (6) technical college/first Bologna cycle, (7) university degree/second degree from Bologna (Bologna master’s degree), (8) master’s degree, and (9) doctorate.

The dual career support variable (Taylor, 2015) is based on the gaps in athletes’ education and employment regarding their sports career to reach the potential of an individual athlete. Therefore, their financial support was measured. According to our previous research (Robnik et al., 2017), successful DC support includes financial support for their sports career and education. This questionnaire comprises seven items measuring DC support programs (Cronbach’s α = 0.86). Athletes were asked if, as part of the DC programs of athletes, they were included in the following DC support programs, including (1) sports scholarships, (2) tutorship program, (3) cofinancing of tuition, (4) Distance Study Program, (5) human resources development in sports project, (6) Professional Athlete Career Program, and (7) employment of athletes in public administration. Participants rated the importance of each program using a 7-point Likert-type scale ranging from 1 (strongly disagree) to 7 (strongly agree).

Sports achievements (SPO) are the basis for athletes’ status rights and DC support of individual athletes (Robnik et al., 2017). Best results/sports achievements were measured at the following events (Küttel, 2017): Olympic Games, world championships, overall World Cup/world ranking list, European championships, junior world, and/or national championships. Participants ranked the status of elite athletes during their sports careers in the following categories: (1) Olympic class, (2) world class, (3) international class, (4) perspective class, and (5) national class.

Reasons for sports career termination and the decision to retire from elite sports were measured with the previously validated Athletic Career Termination Questionnaire (ACTQ) (Küttel, 2017). A total of nine items regarding career-end characteristics were selected from 56 questions in the ACTQ. Athletes assessed reasons for sports career termination related to (1) job (e.g., interesting job possibility and desire to work full-time), (2) education (e.g., to continue education and more time required for studying), (3) performance (e.g., unsatisfactory performance; achieved most sporting-goals; deselection; and not qualified for major events), (4) sports-environment (e.g., conflicts with the federation, coach or team-mates, and doping), (5) health (e.g., injury, illness, and burnout), (6) family (e.g., desire to have a family and more time for family or friends), (7) finances (e.g., lack of financial support and need to earn more money), (8) personal reasons (e.g., tired of elite sports lifestyle; wanting a change; and more time for oneself), and (9) motivation (e.g., lack of goals; difficulty in training; and many deprivations). Participants rated the reasons using a 7-point Likert-type scale ranging from 1 (strongly disagree) to 7 (strongly agree).

### Procedure

The research was conducted according to international ethical guidelines, and anonymity was preserved. Former elite athletes from Slovenia were invited to participate with a clear outline of the conditions regarding the online survey. This survey was translated into Slovene and distributed in different ways. The online questionnaire was distributed to the electronic addresses of sports organizations, including national sports federations affiliated with the National Olympic Committee (NOC), requesting to submit the questionnaires to their members (sports clubs) and athletes in their organizations. The online questionnaire was distributed directly through the email addresses of former elite athletes and the email database of athletes included in the programs and projects of the NOC. The online survey link was included a consent statement.

### Data Analyses

Statistical analyses were conducted using the SPSS 24 version software. Descriptive statistics for the dependent variables, i.e., the social-class position of former elite athletes 5 years after their sports career termination (CLASS), and independent variables, i.e., (1) DC support and (2) reasons for sports career termination (ACTQ), were examined in detail along with the education (EDU) and sports achievements status (SPO). Due to the limitation (sample size) of our study, we focused on fewer variables to increase the reliability. First, to explore the relationships between variables, the correlation analysis was performed and Pearson’s correlation coefficients were calculated. Descriptive statistics for variables and Pearson correlation coefficients (from the Pearson correlation matrix) are presented in Table 2. Following the results of significant correlations with small to medium effect sizes (Cohen, 1988) (*p* < 0.05) with the CLASS variable, the multiple regression analysis, and stepwise regression modeling were performed for significant variables (two variables: DC and ACTQ; 16 items) and education (EDU). The stepwise regression modeling was used to maximize the predictive power of our model. Multiple regression analysis coefficients and multiple regression analysis with the stepwise method are presented in Table 3.

**TABLE 2:** Descriptive statistics for variables and Pearson correlation coefficients (from the Pearson correlation matrix).

Variables	Classification	Frequency (*n*)	Mean (SD)	Pearson correlations
**Social-class profile (CLASS)**			4.16 (2.36)	1
	(I) Higher-grade professionals, administrators, and officials; managers in large industrial establishments; and large proprietors	5 (10.2%)		
	(II) Lower-grade professionals, administrators, officials, higher-grade technicians, and managers in small industrial establishments and supervisors of non-manual employees	8 (16.3%)		
	(IIIa) Routine non-manual employees, higher grade (administration and commerce)	14 (28.6%)		
	(IIIb) Routine non-manual employees and lower grade (sales and services)	2 (4.1%)		
	(IVa) Small proprietors, artisans, etc., with employees	4 (8.2%)		
	(IVb) Small proprietors, artisans, etc., without employees	8 (16.3%)		
	(IVc + V + VI) Skilled workers and small-time owners	2 (4.1%)		
	(VIIa) Semi-skilled and unskilled workers	3 (6.1%)		
	Unemployed	3 (6.1%)		
**Dual career (DC) support**				
	Sports scholarships (1–7)		1.82 (1.93)	0.12
	Tutorship program (1–7)		1.51 (1.37)	0.21
	Co-financing of tuition (1–7)		1.70 (1.69)	0.21
	Distance Study Program (1–7)		1.31 (1.16)	0.17
	Human resources development in sports (1–7)		2.00 (2.03)	0.19
	Professional Athlete Career Program (1–7)		1.84 (1.68)	0.24
	Employment of athletes in public administration (1–7)		1.86 (1.93)	0.35*
**Reasons for sports career termination (ACTQ)**	Job-related reasons (1–7)		2.87 (2.46)	–0.06
	Education-related reasons (1–7)		2.43 (1.86)	–0.04
	Performance-related reasons (1–7)		3.38 (2.47)	0.07
	Sport environmental-related reasons (1–7)		2.80 (2.24)	0.24
	Health-related reasons (1–7)		3.95 (2.52)	–0.08
	Family-related reasons (1–7)		3.50 (2.17)	0.19
	Financial-related reasons (1–7)		3.68 (2.39)	0.38*
	Personal reasons (1–7)		3.39 (2.10)	0.19
	Motivational-related reasons (1–7)		2.73 (2.08)	0.13
**Education (EDU)**			6.14 (1.47)	0.32*
**Sport achievements (SPO)**			2.37 (0.95)	–0.12

Pearson correlation coefficients between variables CLASS and DC support variable: Employment of athletes in public administration (r = 0.35); ACTQ variable: Financial-related reasons (r = 0.38); and EDU variable (r = 0.32). * Correlation is significant at the 0.05 level (two-tailed).

**TABLE 3:** Multiple regression analysis coefficients and multiple regression analysis with the stepwise method.

Methods						Coefficients					
**(1) Multiple regression analysis**											

**(a) Multiple regression analysis coefficients**											

**Model**	**R**	**R-Square**	**Adjusted R square**	**Std. error of the estimate**	**R-square change**		**F-change**	**df1**	**df2**	**Sig. F Change**	**Durbin-Watson**

1	0.387^a^	0.150	0.129	2.22900	0.150		7.239	1	41	0.010	
2	0.511^b^	0.261	0.225	2.10357	0.111		6.035	1	40	0.018	1.978

**(b) Multiple regression analysis with the stepwise method**											

**Model**	**Unstandardized β**		**Unstandardized std. error**		**Standardized β**			***t***		**Sig.**	

1 (Constant)	2.778		0.639					4.349		0.000	
Financial-related reasons	0.389		0.144		0.387			2.691		0.010	
2 (Constant)	6.121		1.489					4.112		0.000	
Financial-related reasons	0.418		0.137		0.417			3.054		0.004	
Education	–0.554		0.226		–0.335			–2.457		0.018	

*Multiple regression analysis (stepwise method) coefficients: ACTQ construct predictors. (Constant): Financial-related reasons and education. Dependent variable: Social class (CLASS). [F(2,40) = 7.082, p-value = 0.002]; R^2^ = 0.26; std. error = 2.10.*

Second, the confirmatory factor analysis was performed. Due to the small sample size, partial least squares path modeling (PLS-PM) was conducted (Hair et al., 2011; Sanchez, 2013) to identify whether the measured variables evaluated the latent variables. Using the PLS-PM method, complex relationships between observed and latent variables illustrating a measurement model and several latent variables representing a structural model were studied. The R Studio (R version 3.5.2) software (Crawley, 2012) was used to implement the PLS-PM method, using the R-package “plspm” (Sanchez et al., 2013, 2017). PLS-PM coefficients and analysis are presented in Table 4.

**TABLE 4:** Partial least squares path modeling (PLS-PM) coefficients and analysis.

Methods	Coefficients					
**(2) PLS-PM analysis**						

**1. Outer model**	**Mode**	**MVs**	**Cronbach’s alpha**	**Dillon-Goldstein’s rho**	**eig.1st**	**eig.2nd**

Dual career support (DC)	A	7	0.86	0.89	3.78	1.42
Education (EDU)	A	4	0.9	0.93	3.06	0.54
Reasons for sports career termination (ACTQ)	A	2	0.29	0.74	1.17	0.83
Social class profile (CLASS)	A	1	1.0	1.0	1.0	0.0

**2. Inner model**	**Estimated value**	**Std. error**	***t*-value**	**Pr(>|t|)**		

Dual career support (DC)	0.344*	0.165	2.09	0.044		
3. Path coefficients matrix						
4. Latent variable scores						
5. Cross-loadings						

**6. Summary inner model**	**Type of variable**	**R^2^**	**Communality**	**Redundancy index**	**AVE**	

Dual career support (DC)	Endogenous	0.22	0.54	0.12	0.54	
Education (EDU)	Endogenous	0.57	0.77	0.44	0.77	
Reasons for sports career termination (ACTQ)	Endogenous	0.27	0.58	0.15	0.58	

**7. Total effects**	**Direct**	**Indirect**		**Total value**		

Dual career support (DC) → Social class profile (CLASS)	0.3442*	–0.00423		0.3400		
8. Unidimensionality						

**9. Goodness-of-fit**	**40% (GoF = 0.4042)**					

10. Bootstrap results						
11. Data matrix						

PLS-PM analysis coefficients–DC construct: t-value = 2.09; p-value = 0.04 (*p < 0.05); R^2^ = 0.22; std. error = 0.17; GoF = 0.40.

## Results

### Social Class Profile of Former Elite and Olympic Athletes

Results showed that the education of former elite athletes included in the research (*n* = 49) was mostly achieved at the university degree/second-degree from Bologna (Bologna master’s degree) (49%) level. Two athletes (4.1%) reached the doctorate level. All former elite athletes reached at least a secondary (22.4%) or vocational (2%) education level. The most represented sport status categories of former elite athletes achieved during their sports careers were international-class (38.8%), followed by world-class (32.7%), and Olympic class (20.4%).

Regarding the category of work, the CLASS of former elite athletes in their post-sport career employment was most represented in the (IIIa) category, routine non-manual employees, and higher grade (administration and commerce) (28.6%). Between (I) higher-grade professionals, administrators, and officials, and managers in large industrial establishments, there were two Olympic and world-class athletes and three international-class athletes (10.2%). Among the CLASS categories, (II) lower-grade professionals, administrators, officials, higher-grade technicians, and managers in small industrial establishments, and (IVb) small proprietors, artisans, etc., without employees, were presented with former elite athletes (16.3%). While (IVa) small proprietors, artisans, etc., with employees, presented (8.2%), three (6.1%) were unemployed former elite athletes.

In the DC support variable, former elite athletes stressed the importance of successful coordination of sport and education for the programs: human resources development in sports (*M* = 2.00, SD = 2.03), followed by employment of athletes in public administration (*M* = 1.86, SD = 1.93), Professional Athlete Career Program (*M* = 1.84, SD = 1.68), and sports scholarships (*M* = 1.82, SD = 1.93).

The perceived reasons for sports career termination (i.e., ACTQ) of most athletes were health-related (*M* = 3.95, SD = 2.51) and finance-related (*M* = 3.68, SD = 2.39). This was followed by family-related (*M* = 3.5, SD = 2.17), personal (*M* = 3.39, SD = 2.1), and performance-related reasons (*M* = 3.38, SD = 2.47).

The correlations were significant between dependent variable CLASS and independent variables, including EDU (*r* = 0.32, *p* < 0.05), DC support for employment of athletes in public administration (*r* = 0.35, *p* < 0.05), and sports career termination (ACTQ) due to financial-related reasons (*r* = 0.38, *p* < 0.05). We also found that education was correlated with the sport status (SPO) of elite athletes (*r* = 0.53, *p* < 0.01).

### Reasons for Termination of Sports Career

The results of the multiple regression analysis and stepwise regression modeling showed that education (EDU) (β = –0.34, *p* < 0.05) and financial-related reasons for sports career termination (ACTQ) (β = 0.42, *p* < 0.05) can contribute to the quality of post-sport career transition.

In the first phase of the stepwise regression modeling, significant variables (two variables: DC and ACTQ; 16 items) and education (EDU) was entered, in a stepwise criterion (Probability-of-F-to-enter ≤ 0.050, Probability-of-F-to-remove ≥ 0.100). The multiple correlation coefficient R in Model 2 indicated a good level of prediction (*R* = 0.511). *R*-squared provides an estimate of the strength between our model and the response variable. The regression model with financial-related reasons and education of predictors accounted for 26.1% of the variance of the social class (CLASS) of former elite athletes. Durbin-Watson scores were close to two (D-W = 1.98); therefore, no autocorrelation existed (Miles and Shevlin, 2001). The overall *F*-test determined that the relationship between our model and response variable was significant [*F*(2,40) = 7.082, *p*-value = 0.002]. The *p*-value of model 2, financial-related reasons (*p* = 0.004), and education (*p* = 0.018) indicated that the null hypothesis for the social class (CLASS) can be rejected.

### Support for Dual Career

The results of the PLS-PM showed that DC support (β = 0.34, *p* < 0.05) can contribute to the quality of post-sport career transition.

The DC latent variable included sports scholarships, tutorship programs, cofinancing of tuition, Distance Study Program, human resources development in sport project, Professional Athlete Career Program, and employment in public administration, and from these, only the last variable was significant (*r* = 0.35, *p* < 0.05) with CLASS. After running the PLS-PM analysis, an assessment of the model was conducted. Assessing the model requires an analysis and interpretation of the measurement model (Cronbach’s α = 0.86; Dillon-Goldstein rho = 0.89, loadings = 0.6-0.8; eig.1st = 3.78) and the structural model [*t*-value = 2.09; std. error = 0.17; *p*-value = 0.04 (*p* < 0.05) *R*^2^ = 0.22 redundancy index = 0.12; AVE = 0.54] (Chin, 1998; Sanchez, 2013). Predictive power of the final PLS-PM model is 40% (goodness-of-fit = 0.40).

## Discussion

The study aimed to examine the impact of factors directly contributing to the quality of post-sport career transition of former elite and Olympic athletes and their social-class position after their sports career termination. The results contributed to the knowledge regarding the mechanism of successful DC support and the quality of post-sport career transitions. First, more studies focus on DC of athletes and related factors, but they were not directly studied regarding the performance in their post-sport career employment. Therefore, in contrast to previous studies (Stambulova and Wylleman, 2019; Fuchs et al., 2021; Moreno et al., 2021), which have addressed the study-sport combination, this study includes the work-sport combination, specifically, the socioeconomic status of retired elite athletes in their post-sport career employment in which the social class concept was used (Erikson and Goldthorpe, 1992; Smith et al., 2013). Second, DC support practice in Slovenia showed that although athletes often have the appropriate education levels, which directly shows successful DC mechanism in education, it does not guarantee successful employment. The DC support mechanism (Taylor, 2015) should support the gaps in athletes’ education and employment regarding their sports career to reach their potential. According to our previous research (Robnik et al., 2017), the mechanism of successful DC support includes financial support for their sports career and education. Third, sports achievements are the basis for the DC support and athletes’ status rights and are directly linked to the DC support of an individual athlete (Robnik et al., 2017). Küttel et al. (2017) compared the athletic retirement of former Swiss, Danish, and Polish athletes and explored the influence of factors on the quality of post-sport career transition. The reasons among former elite athletes from Slovenia are consistent with this study, although the DC support mechanism was different and included the employment of athletes in public administration.

The results showed that education and DC support related to finances can contribute significantly to the quality of post-sport career transitions. The results are consistent with the previous studies (Küttel, 2017), where financial support athletes receive from their federation or the national sport governing body (De Bosscher et al., 2015) and the institutionalized support for athletes’ DC efforts (Aquilina and Henry, 2010, 2014; Stambulova and Ryba, 2013) have a considerable impact on preconditions for the transition. Regarding educational status, results supported previous studies that underlined the importance of education in the quality of post-sport career transitions (Cecić Erpič et al., 2004; Guidotti et al., 2015) and athletes’ retirement processes (Tshube and Feltz, 2015). Results of the sports career termination showed that former elite athletes who ended their sports career due to financial reasons (e.g., lack of financial support, need to earn more money) rank lower on social-class positions. Nationality and culture play a crucial role in the post-sport career employment of elite athletes (Alfermann et al., 2004). Therefore, for the interpretation of results, it is important to acknowledge the national context and specifics (Küttel, 2017; Küttel et al., 2017). This study can confirm that 5 years is a sufficient period for athletes to observe signs of progress in their post-sport career employment and to (a) reassess values, (b) choose a new area of operations, and (c) show positive results in their post-sport career employment. Moreover, the social-class positions of the former elite athletes were measured. The concept of social classes is complex enough to present a comprehensive picture of individuals on a hierarchical level within the society and refers to education (Erikson and Goldthorpe, 1992; Smith et al., 2013). Although the education level of former elite athletes was relatively high [e.g., university degree or second degree from Bologna (Bologna master’s degree)], their social class status was not according to their potential for a career (Stambulova, 2010) (e.g., educational status, competences, sports career success), which would enable them to transition successfully into their post-sport career employment. However, the results of other studies (Küttel et al., 2017) suggest that the socio-cultural context influences the usefulness of these social capital types related to the previous sports career when athletes try to transition to the labor market. This could be because elite athletes in their sport-career termination do not have sufficient working experience equivalent to the requirements of the labor market. Therefore, if an athletic career appears to be just one part of the life career, which concerns athlete’s future when retiring from sport (Zafeiroudi et al., 2020), it is indeed of great importance to be well prepared for development in other spheres of life and to reach potentials of the individual. For future studies, the authors could include athletes’ working experiences in their research. We also measured the socioeconomic status of the former elite athletes with social-class positions. Moreover, when studying (former) international elite athletes, authors should consider that sample size issues are common; therefore, it might be difficult to include a sufficient number of athletes into the study (Küttel, 2017). Further research is needed to explore the pathways athletes take when they leave university (Vickers and Morris, 2021) and how we refer to people engaging in DC (Geary et al., 2021).

In general, the results of this study indicate that data from Slovenian former elite athletes can add value to the research and further development of a proper mechanism of DC support and the quality of post-sport career transitions in an international context. Development of society through positive values of sports is possible, especially with good governance in sports and innovative educational approaches. To achieve these objectives, it is important to establish mechanisms supporting the integrated development of athletes as comprehensive personalities and solving the issues of athletes’ status in their post-sport career employment.

## Conclusion

Understanding how different factors contribute to the integrated development of individual athletes to reach their potential in sports, education, and their post-sport career employment is important for theorists, DC practitioners, and stakeholders working with DC athletes. To develop a sufficient mechanism, DC support providers should consider supporting education along with the financial support of athletes during their sports careers and recognizing study-training ecosystems, based on good practices (Ferjan et al., 2021) to successfully transition to their post-sport careers. These findings can also be useful for athletes and their athletic triangle support network (e.g., coaches and parents) as a support in the decision-making process (Wylleman and Lavallee, 2004; Hakkers, 2019).

## Data Availability Statement

The raw data supporting the conclusions of this article will be made available by the authors, without undue reservation.

## Author Contributions

All authors listed have made a substantial, direct, and intellectual contribution to the work, and approved it for publication.

## Conflict of Interest

The authors declare that the research was conducted in the absence of any commercial or financial relationships that could be construed as a potential conflict of interest.

## Publisher’s Note

All claims expressed in this article are solely those of the authors and do not necessarily represent those of their affiliated organizations, or those of the publisher, the editors and the reviewers. Any product that may be evaluated in this article, or claim that may be made by its manufacturer, is not guaranteed or endorsed by the publisher.
